# Fibrous Body in the Womb

**Published:** 1847-01

**Authors:** 


*Fibrous Body in the Womb.*—Dr. Michom presented the uterus of a woman who had lately died in his wards. When she was admitted the organs were examined, and the toucher detected in the os uteri a solid tumour, to which the floodings the patient complained of were naturally referred. Removal of the fibrous growth was comtemplated, when accurate thoracic disease occurred, and the woman died. On dissection, a fibrous tumour was found in the upper part of the anterior wall of the womb, and the morbid production of the os tincæ contained fluid. M. Michom observed that the error of diagnosis he fell into was almost inevitable, inasmuch as a fibrous production really existed in the womb, causing the losses of blood which had been erroneously attributed to the tumour of the neck.—*Ibid.*

